# Dark Participation in Games

**DOI:** 10.3389/fpsyg.2020.598947

**Published:** 2020-11-10

**Authors:** Rachel Kowert

**Affiliations:** Independent Researcher, Take This, Seattle, WA, United States

**Keywords:** video games, trolling, toxic, online games, dark participation

## Abstract

With the advent of digital games came the advent of gamer cultures and identities. A “gamer” became a new social first for the group of individuals who played video games (primarily in arcades) in the late 1970’s. Over time, however, gamer cultures have grown into what is largely discussed as “toxic cultures,” and come to become more associated with exclusion than inclusion if you don’t fit a certain mold. Despite its prevalence, deviant behaviors in games as a subject of academic study is a confusing space, with different researchers using different criteria to describe the same things. This article provides the first comprehensive cataloging and overview of dark participation in games. This includes defining these behaviors, cataloging their variants, and discussing their social and psychological impact and their potential underpinnings. It is critical to establish a shared language about what these behaviors are in order to effectively understand and combat them.

With the advent of digital games came the advent of gamer cultures and identities. A “gamer” became a new social category for the group of individuals who played video games (primarily in arcades) in the late 1970’s. As part of a socio-cultural niche, “gamer” was established to define and unite this group of people. However, with the popularization and growth of games themselves, the term “gamer” has also grown and changed. Today, this term not only represents players but differentiates “gamers” as their own subcultural movement (Golding, 2014; Grooten and Kowert, 2015). That is, all gamers are players, but not all players are gamers. Today, “gamer” is part of everyday jargon and widely integrated into personal, social, and cultural identities (Grooten and Kowert, 2015). We express our “gamerness” with our choice of clothing, the references we make, and how we view ourselves, others, and our place in the world.

Over time, however, gamer cultures have grown into what is largely discussed as “toxic cultures” and come to become more associated with exclusion than inclusion if you don’t fit a certain mold. This shift was noted by Golding in 2014 with his op-ed “The End of Gamers.” In it, he discussed the shifts in video game cultures away from inclusivity and toward combativeness. Four years later, these same sentiments were discussed by Condis (2018) *New York Times* op-ed:

“As events like the 2014 harassment campaign #GamerGate amply demonstrated, to some members of the gaming community, the increased visibility of people of color, women, and LGBTQ people in gaming circles is seen less as an expansion and more as a hostile takeover.”

Similarly, in a Wu (2019) article, Brianna Wu states:

“Though the gaming world is huge and diverse, and full of smart and wonderful players, it is also thronged by misogynists and racists who feel free to advocate harm against anyone who’s not like them.”

These communities of exclusion and hostility have come to be housed under the umbrella of “toxic gamer cultures,” a phenomenon that is quite well known in gaming communities. At its root, the idea of “toxic gamer cultures” refers to prevalence of deviant behaviors within games but also readily dismissing one’s responsibility for them under the shared idea that it is just part of the “anonymous and toxic gamer” collective identity (Tang and Fox, 2016). Some researchers have gone so far as to predict that the normalization of these behaviors within gamer culture could eventually shift and/or sustain cultural norms toward eventually seeing harassment in-games as harmless and acceptable (Ross and Weaver, 2012; Page et al., 2016).

## Underpinnings of Toxic Gamer Cultures

Why toxic gamer cultures have developed is a question many scholars have attempted to answer. Research has pointed to several environmental, community, demographic, and game play factors that contribute to the creation and sustainment of toxicity in games and gamer cultures. These are discussed in more detail below.

### Environmental and Community Factors

There are several environmental and community factors to consider when discussing toxic behavior in online spaces and, specifically, within online games. Kordyaka and colleagues discuss three of these—social cognitive theory, theory of planned behavior, and the online disinhibition effect—in their 2019 article entitled “Toward a unified theory of toxic behavior in games.” Put briefly, social cognitive theory argues that individuals learn toxic behaviors in games through social learning (Bandura, 1986). That is, any pre-existing toxic gamer cultures within games would perpetuate toxicity. Theory of Planned Behavior (Ajzen, 1991) argues that an individual’s intentions to engage in toxic behavior is based on the specific context of the situation. That is, people may behave negatively toward others if it is accepted as a group norm (i.e., in toxic gamer cultures) and if those who perpetuate toxic behavior do not experience consequences for their actions. Lastly, the authors discuss the online disinhibition effect (Suler, 2004), which is the idea that while you are interacting on the internet, others cannot see you (you are invisible) and that they don’t know you (you are anonymous). This anonymity and invisibility are generally thought of as being among the primary driving forces of toxic behavior because they create an ideal space for people to push social boundaries with a sense of few repercussions. For example, research has found that when anonymity was removed from social media sites, it reduced the amount of trolling behavior (Wright, 2013). Kordyaka and colleagues concluded that the most meaningful predictor of toxic behavior was the online disinhibition effect; however, Social Learning Theory and the Theory of Planned Behavior could play a role in sustaining toxic cultures once they have been established.

An additional factor to consider is the Social Identity Model of Deindividuation Effects model (SIDE; Postmes et al., 1998), which suggests that deindividuation or depersonalization of group members can emphasize the presumed similarities of members within a group and encourage behavior consistent with the group norms. That is, the more anonymous a person is, the more deindividuated they are (i.e., the online disinhibition effect), the more likely they are to adhere to group norms. Research has found support for these ideas, specifically in the realm of toxic behaviors. Amiot et al. (2017) study by Amiot and colleagues found that in-group norms that favor derogatory behaviors toward an “out-group” can predict the likelihood of a member of that group exhibiting those behaviors. That is, if you consider yourself part of the “in-group” (e.g., a gamer) and engage with someone whom you consider part of the “out-group” (e.g., not a gamer) and the in-group norm is toxic behavior toward the out group (e.g., flaming, griefing, and doxing), you are more likely to engage in that kind of behavior. Research from Hilvert-Bruce and Neill (2020) further support this idea, as they found that normative beliefs about cyber aggression among gamers significantly predicted cyber aggression toward other players. This kind of accordance with group norms has also been found in other kinds of online spaces. For example, Zhao et al. (2008) found Facebook users tend to stress their group over personal identity when discussing the ways that they behave online.

Taken together, it seems that the effects of the online disinhibition effect make gaming spaces more open for toxic behavior to happen, with the SIDE (Postmes et al., 1999) effects of the social environment (i.e., the “toxic gamer cultures”) potentially fueling a perpetuating cycle.

### Personality, Gender Socialization, and Age

There are also a range of personality and social factors that have been found to have significant relationships with toxic behavior among online game players. Hong and Cheng (2018) found that social extraversion, a sense of inferiority to others, and depression positively predicted online trolling behavior. Toxic behavior has also been positively correlated with sadism (the tendency to derive pleasure from inflicting suffering), psychopathy (a personality disorder characterized by persistent antisocial behavior and impaired empathy), and Machiavellianism (a personality trait which sees a person so focused on their own interests and goals they will manipulate, deceive, and exploit others to achieve their goals). Of these, the researchers found sadism to have the strongest correlation with toxic behavior and concluded that “online trolling seems to be an internet manifestation of everyday sadism” (Buckels et al., 2014; p. 1). Wai Yen (2020) also found that people who harass other people score higher on measures of Machiavellianism, psychopathy, and gamer identification measures. The relationship with gamer identification would give credence to the suggestions of the SIDE model in gaming spaces.

There’s also the idea that games are a “boy’s toy,” and the toxicity stems from the idea that games are being infiltrated by anyone who does not fit this mold. This can create heightened tension and lead to the harassment of others as a way to make them “leave their space,” so to say (Lucas and Sherry, 2004; Kowert et al., 2017).

Research has also found that age is inversely related to toxicity, with younger players perceiving many forms of dark participation, such as flaming, as less serious or even normal (Mattinen and Macey, 2018).

The individual motivations of the perpetrators of these kinds of behaviors also need to be considered. Cook et al. (2018) interviewed perpetrators of toxic behaviors (i.e., so-called “internet trolls”) to uncover the antecedents for their actions in games specifically. They found that the motivation for these behaviors span three broad categories: attack, sensation seeking, and interaction seeking. Even so, the researchers note that these elements are not mutually exclusive (Cook et al., 2018). Attack focus behaviors are defined as a direct attack on another players’ enjoyment of the game or gameplay. This was the most commonly reported motivation. Sensation-seeking focus refers to behaviors that lead to enjoyable consequences for the troll but are not inherently good or bad for other players (e.g., spamming). Interaction-seeking focus emphasizes trolling as an unorthodox method of communication that the trolls enlist to make players get involved in the conversation and the game. Interaction seeking plays on the idea that “no attention is bad attention.” The researchers also found that the primary trigger for becoming a perpetrator of toxic behavior was social (i.e., if they were trolled themselves). Other triggers included internal (personal enjoyment) and circumstantial.

### Game Play Factors

There are also factors to consider relating to game play itself. Specifically, Cook (2019) notes that imbalance between the skill levels of the players and the challenge of the game (the game is too easy or too frustrating) may be a driving force for toxic behaviors.

The competitive and multiplayer nature of games can also contribute, especially when it comes to verbal forms of dark participation. Work from Hilvert-Bruce and Neill (2020) found that gamers report aggression to be more acceptable and tolerable when it occurs online than offline. Zubek and Khoo (2002) note that when gameplay is more about competition than cooperation, the social environment is more characterized by competitiveness, trash-talking, and gloating. Shores et al. (2014) found that players who choose to play more competitively scored higher on a toxic behavior measure than those who chose to play less competitively. Additionally, Adachi and Willoughby (2011) found that competitiveness in video games was more related to aggressive behavior than violent content of games. Games with competitiveness were found to produce higher levels of aggressive behavior from the players regardless of whether or not the game contained violent content.

## Prevalence and Impact of Toxic Gamer Cultures

While understanding why this behavior takes place is one side of the coin, understanding its prevalence and impact is the other.

A 2019 study by Cary and colleagues found that 80% of players said that they believed the average gamer makes prejudiced comments while playing online. A 2019 report from the ADL reported that 74% of adults who play online multiplayer games in the US experience some form of harassment while online. Cary et al. (2020) found over half of their surveyed players (53%) said they experienced harassment because of their race/ethnicity, religion, ability, gender or sexual orientation and 65% had experienced some form of severe harassment, including physical threats, stalking, and sustained harassment. They also found that nearly 1 in 3 (29%) of players have been doxxed (which is where personal identifiable information is posted publicly online, such as your address and phone number). Taken together, this suggests that more than half of all players have experienced some form of harassment while playing online and suggests that toxic gamer cultures have permeated gaming environments and communities to a substantial degree.

Research has also found that these behaviors in online gaming spaces, however brief, can cause psychological harm to the intended victim and any third-party onlookers who might witness it (de Mesquita Neto and Becker, 2018). In 2019, the ADL found that 1 in 10 game players reported having depressive or suicidal thoughts as a result of harassment in online multiplayer games. Nearly 1 in 10 (8%) reported having to take steps to reduce the threat to their physical safety. The 2020 Byter report indicated that over half of male and female gamers have experienced abuse in games, and nearly a third (28%) reported they experienced it regularly. They also reported that 1 in 4 female gamers reported that the “widespread toxicity” in games made them feel upset, intimated, and made them not want to play again. Heightened anxiety and lower self-esteem has also been reported as a result of victimization within online games (Ewoldsen et al., 2012).

## State of the Research: Dark Participation, Toxicity, and Trolling

Despite its prevalence, deviant or toxic behaviors in games as a subject of academic study is generally a confusing space. Researchers have typically assessed “toxicity” as a broad and general term with little differentiation between different kids of toxic behaviors (e.g., trash-talking, flaming, doxing, etc.). To add to the confusion, many researchers using different criteria to describe the same things (for an overview, see Ortiz, 2020). While some researchers treat any deceptive action online as toxic (Buckels et al., 2014), deception is not always required by other researchers (Fichman and Sanfilippo, 2015). Other negative behaviors with a perceived hostile intent are also sometimes grouped into trolling, while other researchers treat them as separate phenomena, such as griefing and flaming (O’Sullivan and Flanagin, 2003; Coyne et al., 2009; Thacker and Griffiths, 2012). To add to the confusion, the words “trolling” and “toxic” have often been used interchangeably, with little differentiation made to distinguish the two concepts.

There have been a few attempts to catalog different types of toxicity in games; however, they have all been limited in their scope and level detail. For example, Cook et al. (2018) note 10 different kinds of “trolling” behaviors in their catalog, although some of the behaviors included do not necessarily imply hostile intent (such as contrary play). A similar approach was taken by Komaç and Çağıltay, 2019; however, they only note nine different “trolling” behaviors. Blackburn and Kwak (2014) provide a list of “toxic” behavior that includes only seven categories, whereas Saarinen (2017) notes only five kinds of “toxic” behaviors. Kordyaka et al. (2019) are the only known authors to attempt to differentiate “toxic” behavior in terms of their presentation or premeditation by noting some behaviors may occur repeatedly or temporarily. However, in their work they describe toxic behavior vaguely as “a behavior generating anger and frustration in players harming communication and contributing to spreading a bad mood” (p. 2487). Additionally, the only examples in the text of what might constitute toxic behavior are “insults to other players” and “spamming.”

The wide variation and inconsistences in the field are likely due to the fact that formally evaluating toxic behavior in games is a relatively a new area of study. Existing studies on this topic are few and far between, and nearly all of them have been atheoretical due to a lack of empirical basis upon which to build any theories (Herring et al., 2002; Shachaf and Hara, 2010; Thacker and Griffiths, 2012).

## Developing a Shared Language

The aim of this article is to clarify the state of the research and generate a new shared language around toxicity in games by redefining toxicity, trolling, and other key terms in this area of research. Having a shared language within the research community is a critical first step for understanding these kinds of behaviors in games, their antecedents and consequences, and unifying scholarly efforts. This is especially important in the context of digital games as they uniquely allow for verbal and behavioral dark participation due to the interactive nature of the games themselves.

### Redefining Key Terms: Dark Participation, Toxicity, and Trolling

**FIGURE 1:**
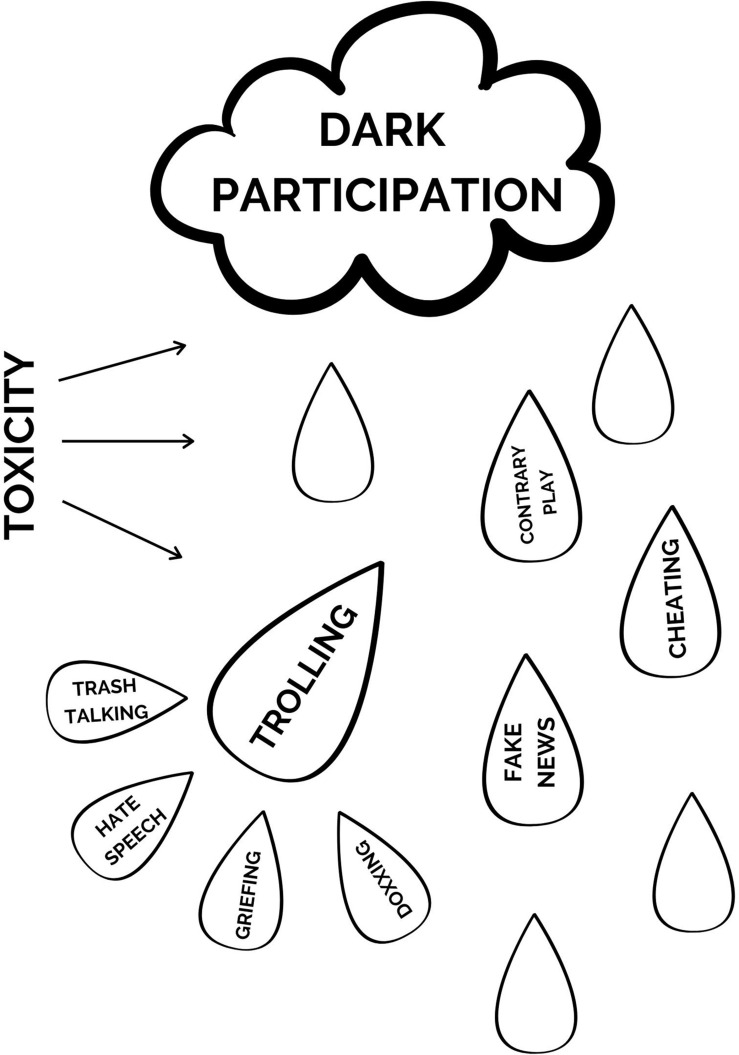
Delineation of dark participation, toxicity, and trolling in online games.

From the broadest perspective, all deviant behavior that takes place online (both in and out of games) can be placed under the broad heading of “dark participation” (Quandt, 2018; see Figure 1). All deviant verbal and behavioral actions that take place on the internet would that fall under this categorization. Any outcome of these behaviors that cause harm to another’s health or well-being (i.e., online propaganda, fake news, harassment, etc.) are considered toxic behaviors.

It is important to note a key distinction between dark participation and toxic behavior in relation to cultural context. Dark participation is *any* deviant action that takes place in online spaces, but what constitutes toxic behavior is often culturally defined (Kwak et al., 2015). Put another way, dark participation is always deviant in the context of the environment, but what behaviors are considered toxic (i.e., behaviors that cause harm to another’s health or well-being) in one situation might not be considered toxic in another. For example, in some circles contrary play (i.e., playing in ways that the game does not intend for you to play) may or may not be considered toxic. Speed running (i.e., completing a game in as little time as possible), for instance, is generally not considered to be toxic even though it is contrary to normal game playing. However, in other cases, using the game in unintended ways could be considered toxic by other players, such as using exploits in World of Warcraft (2004) (Blizzard Entertainment) to gain an unfair advantage. Thus, contrary play is not a behavior that necessarily causes harm or disrupts the play of others but is deviant (i.e., dark participation) and, depending on the context, could be considered toxic.

While toxicity refers to particular *outcomes* of dark participation, trolling refers to the *intent* of the perpetrator. In internet slang, a “troll” is someone who sows discord on the internet with the deliberate intent of eliciting an emotional response or otherwise disrupting on-topic discussions and actions among other players. Deliberate intent being the key phrase in this definition. As seen in Figure 1, toxic behaviors considered to be trolling include actions such as trash talking, griefing, and doxxing as all of these actions are done with the specific intent of causing annoyance, distress, or harm to another player.

### Cataloging Dark Participation in Games

To develop a more comprehensive catalog of what is considered dark participation in games, research literature containing the keywords “toxic,” “trolling,” “dark participation,” and “games” were searched for and retrieved via Google Scholar. Over 50 articles were identified via these search criteria. However, the vast majority of them discussed trolling and toxicity in broad terms, using such definitions as “toxic behavior happens when players break coexistence rules, acting in antisocial ways that brings forth anger or frustration on other players, leading to a bad game experience (Neto et al., 2017, p. 26).” From this collection of work, only eight pieces of scholarly work (seven peer-reviewed articles, one dissertation, and one book chapter) were found identified that had produced a list of different types of dark participation/toxicity in games (i.e., Blackburn and Kwak, 2014; Kwak and Blackburn, 2014; Kwak et al., 2015; Fichman and Sanfilippo, 2016; Neto et al., 2017; Saarinen, 2017; Cook et al., 2018; de Mesquita Neto and Becker, 2018; Komaç and Çağıltay, 2019).

After compiling an initial list of terms from these eight articles, the gaming community was enlisted to make suggestions to the list via social media (i.e., Twitter and Facebook). Drawing from the aforementioned research and community suggestions, a list of toxic behaviors in games was developed and can be seen in Table 1. All of the terms were drawn from the literature with the exception of “swatting,” which was noted by several members of the gaming community as a missing component to the list.

**TABLE 1:** Toxic actions in games and gamer culture from verbal/transient to behavioral/strategic.

	Description	Transient/Strategic
**Verbal actions**		
Trash talking	Putting down or making fun of other players	Transient
Misinformation	Repeating game-unrelated chat	Transient
Spamming (verbal)	Repeatedly engaging in an action, such as sending the same verbal message or using the same in-game move, often to the consternation of others.	Transient
Griefing	Irritating and/or harassing other players by using the game in unintended ways	Transient
Sexual harassment	Insults or comments based on gender, including threats, the criticism of women and their interests, and stalking	Transient
Hate speech	Insults based on religion, ethnicity, nationality, or other personal information	Transient
Threats of violence	Threats of physical abuse, vandalism, arson, sabotage, possession, or use of weapons or other dangerous act	Transient
Flaming	Presenting emotionally fueled or contrary statements with an instrumental purpose	Strategic
**Behavioral actions**		
Spamming (behavioral)	Repeatedly engaging in an action, such as using the same in-game move, often to the consternation of others	Transient
Inappropriate role-playing	Pretending you are a different person to obtain a specific reaction or not abiding by role playing norms of the game and/or community	Strategic
Contrary play	Playing the game outside of what it is intended by most players	Strategic
Inhibiting team	Inhibiting your own team from being successful in winning	Strategic
Aiding the enemy	Behaving in a way that strategically aids the opposing team	Strategic
In-game cheating	Using methods to create advantage beyond normal gameplay in order to make the game easier for oneself	Strategic
Hate raiding	Purposefully infiltrating the gaming space of another with the intention of spreading hate/harassment	Strategic
Doxxing	Publicly sharing and/or publishing another player’s identifying information	Strategic
Swatting	Prank calling emergency services in an attempt to dispatch armed police officers to a particular address	Strategic

**FIGURE 2:**
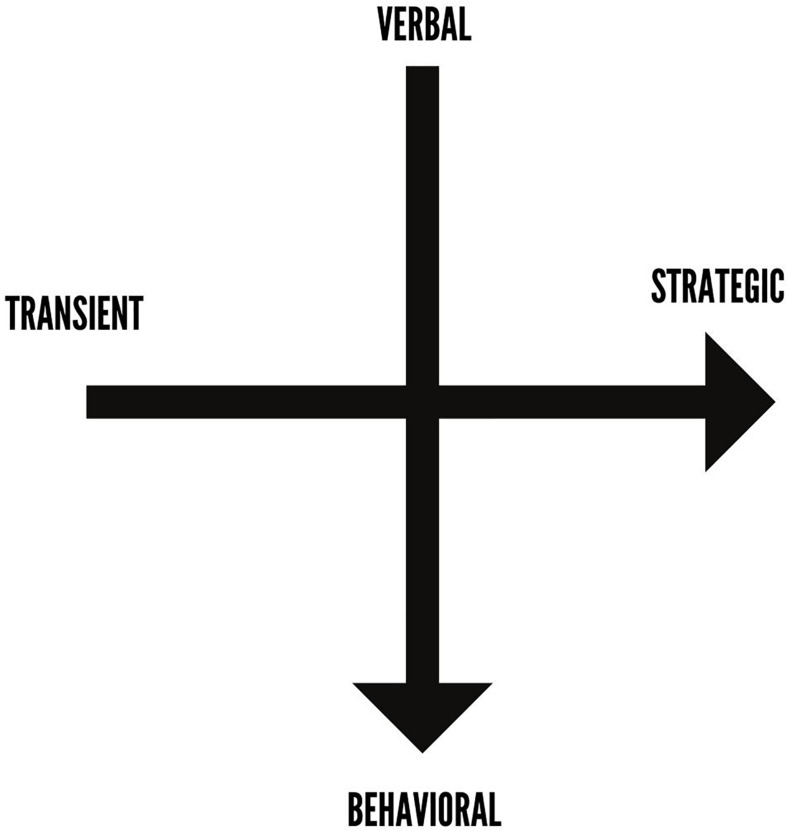
Axes of dark participation in games.

This list also provides the first categorization of dark participation in games across their spectrum of characteristics, verbal to behavioral and transient to strategic (see Figure 2).

A verbal action is one that is expressed verbally (via voice chat or text) from one player to another, whereas a behavioral action is one that is either performed with one’s in-game character or triggers an “out-of-game” action. “Transient” refers to an action that is often committed “in the moment,” whereas “strategic” implies that the individual took time to gather information and formulate a plan before performing the action.

It is important to recognize the difference in behaviors as this can greatly influence the perception of their severity and their impact on the victim of the behavior. For example, doxxing (behavioral, strategic) is more likely to have a severe and long-term negative impact on the victim than trash-talking (verbal, transient). Supporting this, research has noted that trash-talking is less likely a form of “trolling” (i.e., an action with negative intent) and more likely simply a normal by-product of competition, like seen in traditional sports (Türkay et al., 2020). In contrast, doxxing can and has led to long-term psychological challenges, such as post-traumatic stress disorder (Allegra, 2017).

### Subcategories of Dark Participation

It is important to note that the categories of dark participation outlined in Table 1 can (and often do) have subcategories nested within them. For example, as noted by Kwak et al. (2015), various forms of “inhibiting team,” such as refusing to communicate, leaving the game/going AFK, or being an unskilled player. Similarly, Saarinen (2017) subdivides “griefing” into four categories: harassment, power imposition, scamming, and greed play. Cataloging all the variants that may fall within the different categories identified in Table 1 is not within the scope of the current work. The aim of the classification system outlined in this article was to catalog the higher-order categories of dark participation in games. Future work should consider identifying the different subcategories of behavior in games to provide a more comprehensive understanding of the spectrum of behaviors.

## Moving Forward

Understanding what toxicity is, why it happens, and its prevalence within gaming communities is the first step to understanding how to combat it. For example, we know that toxic behavior is largely driven and sustained by anonymity and disinhibition, and a lack of accountability means we can make change by increasing accountability through more effective in-game reporting systems. The Anti-Defamation league [ADL] (2019) reports that 62% of players think companies should do more to make online games safer and more inclusive. Game companies could also enlist more specific guidelines to curate community building. Tran (2019) article by Victoria Tran discusses how specificity in community design can help foster less toxic, more inclusive communities.

More research collaborations are also needed between the industry, academia, and organizations. While many companies do in-house research, that information is largely proprietary. While proprietary information may help one company, sharing that information and engaging in collaboration can help entire communities. We need more transparency and concerted efforts to understand toxic behavior and how to effectively address it. For example, textual analysis such as the ones done by Kwak and Blackburn (2014), Neto et al. (2017), and de Mesquita Neto and Becker (2018), could better help to predict when the more severe forms of dark participation may begin to occur, particularly in highly competitive in-game scenarios. Cooperation from the video game industry by providing in-game chat logs and or other kinds of server data would help push the development of detection systems for particularly deviant players.

Last but not least, we need to mobilize gamers themselves. Research has found that confronting toxic behaviors in games is one of the most effective ways to extinguish the behavior (Whitty and Carr, 2006; Young and Jordan, 2013; Ridout and Campbell, 2014; Tang et al., 2020). However, recent reports indicate that only 18% (Cary et al., 2020) to 20% of gamers say they stand up to harassment when they see it (D’Anastasio, 2020), even though 76% of players (Cary et al., 2020) felt prejudice should be confronted in online games. Change from the bottom up is also the only way to begin cultural change, which Hilvert-Bruce and Neill (2020) note “modification of beliefs which support the legitimacy and acceptability of cyber-aggression in games” (p. 303) are key to prevention and intervention efforts relating to dark participation in games.

## Concluding Thoughts

Toxic behavior in games is a real problem as over 53% said they experienced harassment because of their race/ethnicity, religion, ability, gender, or sexual orientation and 65% had experienced some form of severe harassment, including physical threats, stalking, and sustained harassment. This article provided the first attempt to comprehensively catalog what constitutes dark participation in games in order to establish a shared language. This shared language is the first step critical step needed to better understand these behaviors and how to combat them.

## Data Availability Statement

The original contributions presented in the study are included in the article/supplementary material, further inquiries can be directed to the corresponding author.

## Author Contributions

RK researched and wrote the article.

## Conflict of Interest

The author declares that the research was conducted in the absence of any commercial or financial relationships that could be construed as a potential conflict of interest.
